# NIA Division of Aging Biology

**DOI:** 10.1093/geroni/igab046.1049

**Published:** 2021-12-17

**Authors:** Ronald Kohanski

**Affiliations:** National Institute on Aging, Bethesda, Maryland, United States

## Abstract

Dr. Kohanski will discuss research priorities for the Division of Aging Biology. Dr. Kohanski will also be available for small group discussion.

